# Effects of Alkaline Cleaning Agents on the Filtration Performance and Aging of Polyvinylidene Fluoride Membranes

**DOI:** 10.3390/membranes16040138

**Published:** 2026-04-01

**Authors:** Marek Gryta, Piotr Woźniak

**Affiliations:** Faculty of Chemical Technology and Engineering, West Pomeranian University of Technology in Szczecin, ul. Pułaskiego 10, 70-322 Szczecin, Poland; piotr.wozniak@zut.edu.pl

**Keywords:** PVDF, ultrafiltration, fouling, alkaline cleaning, membrane aging, car wash

## Abstract

Polyvinylidene fluoride (PVDF) membranes are used in ultrafiltration systems for car wash water reuse, where frequent alkaline cleaning is required to maintain operational flux rates. Although NaOH-induced degradation of virgin PVDF membranes has been reported, its relevance under real industrial conditions remains poorly understood. This study investigates the long-term exposure of tubular PVDF membranes to alkaline car wash detergents and evaluates how the resulting structural changes influence permeate quality. During several months of pilot-scale operation with synthetic car wash wastewater and daily alkaline cleaning (pH > 11.5), permeate fluxes remained stable at 50–70 LMH despite pronounced membrane aging. Structural analyses revealed enlarged pore size, increased water permeability and reduced dextran retention, while FTIR confirmed dehydrofluorination of the polymer matrix. Despite the extensive degradation of the membrane skin layer, permeate turbidity, dissolved organic carbon, and surfactant concentrations remained stable throughout the operation. This stability was attributed to the persistent fouling layer, which acted as an effective secondary separation barrier and compensated for the loss of intrinsic membrane selectivity. These findings demonstrate that substantial PVDF degradation does not necessarily compromise permeate quality in car wash ultrafiltration systems, highlighting the dominant role of fouling-controlled separation under long-term alkaline cleaning regimes.

## 1. Introduction

Among the polymeric materials used in ultrafiltration (UF) membrane fabrication, polyvinylidene fluoride (PVDF) has gained widespread recognition due to its excellent chemical resistance, thermal stability, mechanical robustness, and ease of processing [1,2,3]. These properties make PVDF membranes particularly suitable for long-term operation under harsh chemical and thermal conditions [4]. However, the intrinsic hydrophobicity of PVDF often leads to membrane fouling, which can significantly impair separation performance and operational efficiency [1,5,6,7]. Nonetheless, these properties can be mitigated by modifying the membrane surface [1,6,8,9,10]. Furthermore, both the extent of fouling and the choice of membrane-cleaning method are strongly influenced by the specific application of the UF process [2,11,12,13,14].

Ultrafiltration and other membrane processes enable water reuse and are therefore increasingly adopted in the car wash industry [15,16,17,18]. Currently, most car washes use reverse osmosis (RO) systems to produce high-quality washing water [19,20]. Car washes are usually supplied with municipal water containing dissolved minerals that can leave spots and residues on vehicles after drying. An RO system filters out these minerals, producing pure water (permeate) that results in a spot-free rinse. Municipal water is pre-filtered and dechlorinated before being fed to RO modules [20]. A more extensive pre-treatment stage is expected when using membranes for wastewater separation [16,17]. Car wash wastewater (CWW) pre-treated with coagulation and sand filtration can be successfully post-treated using ultrafiltration [21]. The degree of purification can be increased by applying biological treatment in addition to coagulation–flocculation and sand filtration [22]. However, multi-stage systems are complex and expensive for the effective treatment of CWW [16]. Therefore, simpler filtration-based solutions have been proposed [23]. Currently, over 50% of car washes have recycling systems that use sedimentation and sand filtration, assisted by water aeration and ozonation [16,19,24].

The type of car wash service determines the applied water recycling system. There are four types of car wash services: hand wash, self-serve wash, tunnel wash, and automatic wash [16,25]. Various methods are used for car washing, such as: brush roll-over systems, touchless roll-over systems, soft cloth tunnel washes, touchless tunnel washes, self-service wash stations with pressure guns, and self-service stations with foam brushes [19]. Small car washes can only implement simple and inexpensive cleaning systems.

Depending on their design and size, car washes use various methods for water recovery and for treating the resulting wastewater. Solutions applied worldwide and the related problems have been presented in several review papers [26,27,28]. In recent years, small self-service stations have become very popular, and there are several thousand of them in Poland. These car washes are only equipped with a sedimentation tank and an oil separator. Such pre-treated wastewater is discharged into the sewer system. With this setup and a municipal water supply, the cost per cubic meter of water used is only $5–7. Under these circumstances, there is little incentive to invest in a complex water treatment system. Consequently, car wash operators and equipment manufacturers increasingly favor simplified water reuse systems, typically comprising a pump and an ultrafiltration module. A concept for such a compact system for CWW treatment employing tubular PVDF UF membranes was presented in [29].

The UF process removes suspended solids, bacteria, and oil contaminants, which is essential for recovering washing water from CWW [16,17,18]. For separating CWW with high turbidity, it is advantageous to use tubular membranes with large open flow channels [29,30,31]. These allow higher solids to pass through the system; however, some of them will still form a filter cake [29]. Additionally, other CWW components, such as oil and detergents, cause fouling, resulting in a systematic decrease in the permeate flux [32,33]. Periodically washing the tubular membranes with a commercial alkaline agent reduced fouling during CWW separation [34]. The P3 Ultrasil 11 agent has been successfully used to clean membranes that have been fouled by oils, fatty acids, resin acids, and waxes [29,32,35]. In addition to NaOH, this agent contains detergents and EDTA, which increase the cleaning efficiency of membranes when fouling and cleaning cycles are repeated [35,36].

Industrial UF installations use cleaning-in-place (CIP) systems for fouling mitigation [37]. These systems can also be used in large industrial car washes [38]. However, due to the cost, they are not feasible for small car washes with limited revenue. Car washes are equipped with systems for preparing cleaning solutions. With minor modifications, these systems can also be used to clean UF modules, significantly reducing investment costs. This concept was presented in previous papers [29,39]. The papers demonstrated that the alkaline cleaning agents used in car washes to clean rims and remove insects (pH > 11.5) were also effective in cleaning polyethersulfone (PES) membranes contaminated by car wash wastewater.

The feasibility of using car wash systems for membrane cleaning was confirmed during UF studies of real car wash wastewater. In these studies, fouling was reduced by washing PES membranes with an alkaline solution (pH = 11.5) for 30 min every 5–10 h [29]. However, alkaline cleaning can cause unfavorable changes in the membrane structure, so it is recommended to use it no more than once per week [30,40]. Alkaline solutions are also effective for cleaning PVDF membranes; however, these membranes exhibit limited resistance to prolonged exposure to NaOH solutions [41,42]. For this reason, presented studies were conducted to evaluate the degradation level of these membranes during CWW treatment.

The NaOH solutions have been found to reduce the crystallinity of PVDF membranes, which can significantly impact their performance [3]. PVDF exhibits a complex crystalline polymorphism comprising three primary phases: α, β, and γ. Among these, the β phase typically predominates within the membrane matrix [3]. Transitioning from the β phase to the α phase can negatively impact the PVDF membrane’s flexibility [6,43]. Additionally, NaOH solutions have been shown to cause dehydrofluorination, a reaction that removes hydrogen fluoride (HF) units from the polymer chain to form a C=C bond [11,41,44]. For these reasons, the interaction of NaOH with the polymeric PVDF matrix can cause membrane degradation.

Degradation studies are typically performed by immersing membranes in NaOH solutions for a short period [3,45]. Based on these results, it was estimated that alkaline washing of the PVDF membrane every 15 days would lead to significant structural changes after approximately 1.8 years [4]. However, under industrial operating conditions, the membrane surface becomes fouled. In this case, the influence of the membrane material itself becomes less pronounced, while interactions between the cleaning agent and the foulant dominate [35]. Therefore, the presented study focused on investigating the degradation of PVDF membranes that were cyclically fouled by treated CWW.

In this work it was assumed that the protective effect of the fouling layer makes it possible to use PVDF membranes for CWW separation with frequent alkaline cleaning. Coating the PVDF surface with a surfactant layer (SDS) was found to significantly increase the membranes’ resistance to NaOH solutions [41]. Car washing cleaning agents contain significant amounts of surfactants that adsorb onto the surface of PVDF membranes [31]. Therefore, it can be expected that the deposition of CWW components on PVDF membrane surfaces should mitigate the negative impact of the alkaline cleaning solutions. However, previous investigations [39] found that after 18 months of UF system operation with P3 Ultrasil 11 as the washing solution, significant structural changes occurred in the PVDF membranes. Therefore, long-term studies are necessary to evaluate potential changes in membrane properties under industrial operating conditions [46]. Considering that the use of alkaline agents designed for car washing may affect PVDF membrane degradation differently than dedicated membrane cleaners, an additional 9-month investigation was carried out using these detergents.

Although NaOH-induced degradation of virgin PVDF membranes has been widely studied [3,45], membrane behavior under real operating conditions can differ substantially due to fouling, surfactant adsorption, and repeated chemical exposure during daily operation. As shown in our previous work, even long-term cleaning with a dedicated alkaline agent (P3 Ultrasil 11) can significantly alter PVDF structure [39]. However, the detergents routinely used in small car wash facilities differ markedly in composition from specialized membrane cleaners, and their long-term effects on membrane integrity remain unknown. This knowledge gap is critical because many low-income car wash operators rely solely on such detergents, making membrane cleaning both economically feasible and operationally realistic.

To address this gap, the present study provides the first comprehensive evaluation of PVDF membrane degradation under prolonged exposure to real car wash detergents, combined with cumulative alkaline cleaning and degradation-accelerating procedures. Unlike previous short-term laboratory tests, this work is based on nearly three years of pilot-scale ultrafiltration using industrial tubular PVDF modules and wastewater of highly variable composition. The study systematically links structural aging processes with performance indicators—including dextran rejection, turbidity, dissolved organic carbon, and surfactant removal—thereby identifying the long-term functional limits of PVDF membranes in car wash water reuse systems. This integrated assessment offers new insights into membrane viability under economically constrained real-world operating conditions and provides practical evidence supporting the implementation of low-cost cleaning strategies in small car wash facilities.

## 2. Materials and Methods

### 2.1. UF Installation

**Figure A1:**
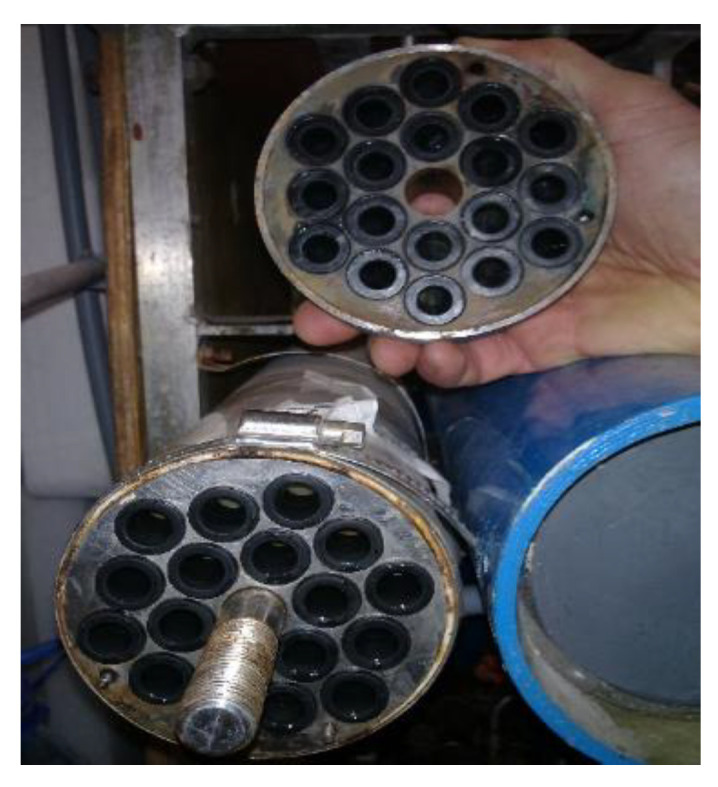
Head section of the tubular module B1.

Laboratory studies have demonstrated the suitability of tubular PVDF membranes for CWW separation [29]. Therefore, the FP100 (100 kDa) tubular membrane (PCI Membranes, Kostrzyn Wielkopolski, Poland), with an internal diameter of 12.5 mm, was selected for pilot plant testing. These membranes are assembled in B1 series modules of various sizes [47]. For this study, a B1 module measuring 1.2 m in length and 0.1 m in diameter was used. The module contained 18 tubular membranes with a total surface area of 0.9 m^2^. A designed end-cap (Figure A1) providing 18 parallel channels and a parallel feed flow arrangement was used, allowing similar process conditions in each FP100 tube.

**Figure 1:**
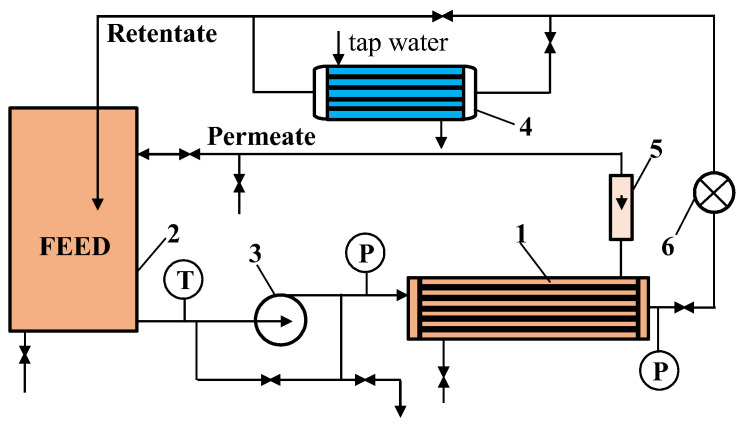
Experimental UF pilot plant. 1—tubular module, 2—feed tank, 3—pump, 4—heat exchanger, 5—rotameter (permeate), 6—flowmeter, T—thermometer, P—manometer.

The pilot plant had a 200 L feed tank connected to a Grundfos (Bjerringbro, Denmark) VNR-8 centrifugal pump feeding a tubular B1 module (Figure 1). UF studies of CWW were performed at a transmembrane pressure (TMP) of 0.1 MPa and a feed flow velocity of 1.9 m/s. Additionally, a series of UF experiments were conducted at higher values of TMP, and maximum permeate flux was measured at a TMP ranging from 0.05 to 0.4 MPa. The permeate and retentate obtained in the UF process were recirculated back to the feed tank, which made it possible to maintain a constant feed concentration. Part of the retentate flowed through a heat exchanger cooled with tap water, allowing the feed temperature to be adjusted. In the conducted UF experiments it was 298 ± 1 K.

The permeate flow obtained was measured using a rotameter (±2 L/h) (Zakłady Automatyki ROTAMETR, Gliwice, Poland) and converted into liters produced per 1 m^2^ within 1 h (LMH). During CWW filtration, the flux decreased, which was mitigated by applying cyclic membrane cleaning. To investigate fouling intensity and the effectiveness of the membrane washing methods used, the tubular membrane was periodically removed from the module as described in Appendix A (Figure A1). For SEM and FTIR analyses, membrane samples were taken 50–60 cm from the tube inlet.

### 2.2. Working Solutions

Car washes are equipped with systems producing softened water, which is used to prepare cleaning solutions. In this study, tap water (500–560 μS/cm) softened by nanofiltration (NF) was used as a processing water. The obtained NF permeate, with a conductivity of 80–90 μS/cm, was used to prepare the feed and cleaning solutions, and was also applied for rinsing the module. The NF permeate was also used as feed for determining the water flux values.

During the experiments, alkaline membrane washing was applied. To compare the effects of different cleaning agents, the resistance of PVDF membranes to alkaline solutions was evaluated using both a professional membrane-cleaning agent (P3 Ultrasil 11, Ecolab, Düsseldorf, Germany) and the alkaline detergents commonly applied in car wash facilities (Table 1).

Deposits that accumulate on the membrane surface reduce the negative effects of NaOH solutions [35]. Therefore, the impact of these solutions on membranes covered with a fouling layer formed during CWW treatment was evaluated. Contaminants removed from cars, along with cleaning agents (surfactants and waxes) used in car washes, contributed to the fouling of UF membranes [32,48]. In this study, cleaning agents supplied by the car wash manufacturer (EuroEcol, Łódź, Poland) were applied. Table 1 shows the composition of the concentrates used to prepare the synthetic CWW. The CWW was a mixture of a 0.5 vol.% foaming solution and a 0.2 vol.% wax solution. The dilutions of the agents used were similar to those applied in car washes. Table 1 also presents the composition of P3 Ultrasil 11 (Hankel Ecolab, Suturamed, Szczecin, Poland). The company PCI Membranes recommends this product for cleaning the FP100 membranes.

Various agents are used for car washing; therefore, when searching for membranes suitable for a car wash, the research cannot be carried out using only a single type of cleaning agent. For this purpose, various synthetic CWWs with compositions given in Table 2 were used in the pilot studies. Previous studies have also shown that dyes added to cleaning agents affect fouling [32]. Moreover, the concentrations of the ingredients in the manufactured concentrates may vary within a certain range (Table 1). For this reason, the properties of CWW3 differed from those of CWW4, which was prepared with the same cleaning agents but produced several months later. For the given type of wastewater used, the UF tests were conducted at constant feed concentrations, with both the retentate and permeate returned to the feed tank. Initially, the feed tank was filled with 100 L of the test solutions.

The pilot installation was located in a university laboratory; therefore, the UF process was not operated during nights and non-working days. Before long-term shutdowns, the installation was rinsed with water and the UF system was preserved according to the manufacturer’s recommendations. For this purpose, the membranes were rinsed using a 0.25 wt% sodium metabisulfite (Na_2_S_2_O_5_) solution (ChemLand, Stargard, Poland).

### 2.3. Membrane Cleaning

During the CWW treatment experiments, the membranes were cleaned periodically. The degradation of the membranes depended on the washing method used, so several different methods were employed:

(a) Flushing: after CWW removal, 100 L of water flowed through the module.
(b) Rinsing: after flushing, the feed tank was filled with 100 L of water that circulated through the system for one hour.
(c) Osmotic rinsing: after step (b), the system was rinsed with water that had remained in the module during the overnight shutdown.
(d) P3 Ultrasil 11 washing: after flushing, the UF installation was washed with a 0.1% solution of P3 Ultrasil 11 (pH = 11.9) for 30 min. Then, the membranes were rinsed with water for 30 min. The solution temperature was typically 293–295 K. This washing procedure is recommended by the membrane manufacturer (PCI Membranes).
(e) Washing with car wash agents: 0.5 vol.% solution of either Euro Insect (pH = 11.5) or Euro Wheel Cleaner (pH = 11.4–11.6) was used. A total of 100 L of cleaning solution was prepared and recirculated through the UF module for 30 min, followed by flushing (a). Additionally, washing tests were conducted with a 60 min wash cycle and with the cleaning solution left in the UF module overnight.
(f) Module maintenance during multi-day breaks: after rinsing with water, the module was washed with a 0.25 wt% sodium metabisulfite solution for 30 min. Then, the solution was removed from the system. After the break, the system was rinsed with 100 L of water, and CWW separation tests resumed.

According to the manufacturer’s instructions, tubular FP100 membranes can be used at pH levels up to 12. For higher pH values (12.8–13.5), NaOH solutions cause significant changes in the PVDF structure of the membranes after just a few hours [49].

### 2.4. Analytical Methods

Fouling and chemical cleaning can alter the separation properties of membranes. To assess this phenomenon, periodic separation studies of dextran, surfactants, and chemical oxygen demand (COD) were conducted. Rejection efficiency (R) was determined as follows:

(1) $$R\;[\%]=\frac{C_{F}-C_{P}}{C_{F}}100$$

where C_F_ [mg/L] and C_P_ [mg/L] are the measured concentrations of the feed and permeate, respectively.

The concentration of dextran was analyzed using a high-performance liquid chromatograph (UlitiMate 3000, Dionex, Sunnyvale, CA, USA) with PolySep-GFC-P 4000 column (Phenomenex, Torrance, CA, USA). For the UF studies, a series of solutions containing 0.05 g/L of dextran with a molecular weight between 100 and 500 kDa (Polfa, Warszawa, Poland) was prepared. The Hach cuvette tests were used to determine the concentration of surfactants (LCK 333—nonionic, LCK 432—anionic), and COD (LCK 1014). The values were recorded automatically with the use of a DR2800 spectrophotometer (Hach Lange, Düsseldorf, Germany). The turbidity of the tested solutions was measured using a model 2100 AN IS turbidity meter (Hach Company, Loveland, CO, USA) with a detection limit of 0.01 NTU.

The degradation phenomenon results in changes in the membrane morphology, which were observed using a scanning electron microscope (SEM, SU8020, Hitachi, Tokyo, Japan), equipped with an EDS system. The functional groups presented onto the membrane surfaces were identified by attenuated total reflection Fourier transform infrared spectroscopy (ATR FTIR) using a Nicolet 380 FT-IR spectrophotometer with Smart Orbit diamond ATR accessory (Thermo Electron Corp., Austin, TX, USA).

## 3. Results

### 3.1. Membrane Properties Stabilization

Changes in membrane performance can result from wastewater treatment, compression, and chemical cleaning. Even after the first chemical cleaning, significant changes to the membrane’s structure occur [44,49]. Therefore, it is important to assess the industrial suitability of membranes once their properties have stabilized. A previous paper presented changes in the performance of FP100 membranes during the initial period of module operation in a UF pilot plant [50]. The results indicated that stabilizing the properties of new membranes after a few days of the UF process is possible by performing at least 2–3 fouling–washing cycles. This provides initial data on membrane properties, which serves as a reference for evaluating changes during subsequent months of UF installation operation.

**Figure 2:**
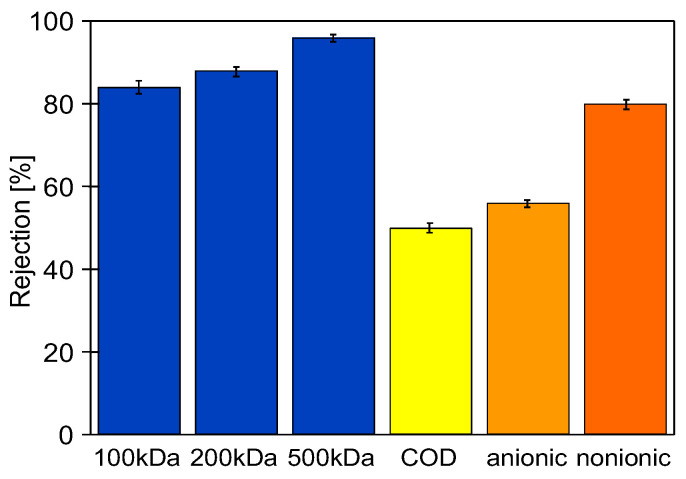
Rejection of dextrans, surfactants (anionic and nonionic), and COD by studied FP100 membranes after the stabilization operation. Feed: dextran solutions (0.05 g/L) or CWW1. TMP = 0.1 MPa.

For the above reasons, a set of operations was performed to stabilize the PVDF membranes at the start of the CWW ultrafiltration studies, including chemical cleaning. For the first three days, the UF module was rinsed with water to remove preservatives from the membranes, and compressed at TMP of 0.2–0.4 MPa. After four days, the system was washed with a P3 Ultrasil 11 solution (pH = 11.9) and rinsed with water. That resulted in a stable permeate flux of 70 LMH (TMP = 0.1 MPa). Next, dextran separation tests were performed achieving 87% retention of 200 kDa dextran and over 96% retention of 500 kDa dextran (Figure 2). The module was then washed with Euro Insect solution (pH = 11.5), resulting in a water flux of 65 LMH (TMP = 0.1 MPa). After washing the membranes with an Na_2_S_2_O_5_ solution for 30 min, the installation was not operated for one week. Then, it was rinsed twice with water, achieving a flux of 72 LMH. After these operations, car wash wastewater separation tests commenced.

After 10 h of CWW1 ultrafiltration, analysis of the feed and permeate composition showed that the rejection of COD was 48%, anionic surfactants 56%, and nonionic surfactants 81%. The permeate turbidity was below 0.15 NTU. Assuming the values presented in Figure 2 reflect initial membrane performance, their changes were analyzed over the subsequent months of FP100 membrane operation in the UF pilot system (Figure 1).

### 3.2. Membrane Fouling

**Figure 3:**
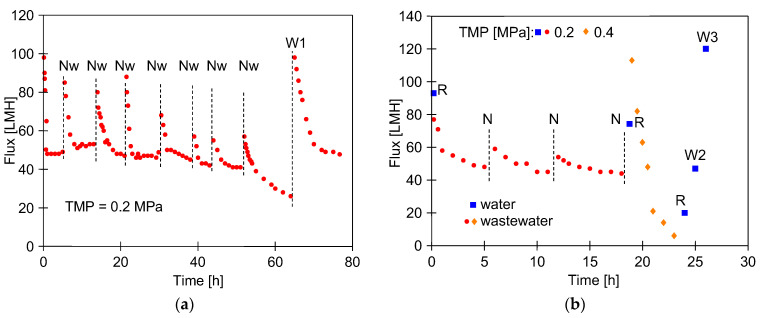
Changes in the permeate flux during treatment of CWW1. During the overnight break, the module was filled: (**a**) Nw—with water, and (**b**) N—with CWW1. W1–W3—membranes washed (30 min) with 0.5 vol.% Euro Insect solution (pH = 11.5). Washing temperatures: W1 and W2—295 K; W3—323–328 K. R—membranes rinsed with water (30 min).

Laboratory-scale studies of FP100 membranes have shown that fouling intensity during the CWW separation depends on both the TMP value and the method and frequency of membrane cleaning [29,50]. Similarly, in the pilot studies, fouling caused a decrease in the permeate flux, which stabilized at 50 LMH during CWW1 treatment at a TMP of 0.2 MPa (Figure 3a). Similar efficiencies were obtained for FP100 membranes during real CWW separation [29].

Water rinsing is a simple method of mitigating fouling that does not cause PVDF membrane degradation. Moreover, the small-scale car washes generally do not operate at night, thus the possibility of utilizing this time for osmotic rinsing was examined. In this case, the membranes were rinsed with water after the UF process, and the system was left filled overnight. After an overnight break, the permeate flux increased above 80 LMH. However, this effect diminished over the following days, and after 64 h of the UF process the permeate flux decreased to 26 LMH. The application of Euro Insect solution (pH = 11.5) for chemical cleaning resulted in the recovery of the initial yield (100 LMH) in this series (Figure 3a, W1). These results suggest that rinsing the modules with water alone does not sufficiently reduce fouling, as has been observed in other UF studies [44,48].

The studied concept of washing water recovery assumed that the UF modules would be fed with CWW pre-treated by the car wash clarifier–degreaser system. In addition to the residues removed from the cars, such wastewater contains cleaning agents [29]. It was investigated whether the agents could clean the membranes during UF plant shutdowns. Studies showed that leaving the wastewater in the module overnight did not result in a significant increase in performance, as was observed with osmotic water rinsing (Figure 3a), and after three days of UF operation, the maximum permeate flux decreased from 93 to 75 LMH (Figure 3b, R points). These results suggest that leaving wastewater in the module for a few hours neither increases nor reduces membrane fouling.

**Figure 4:**
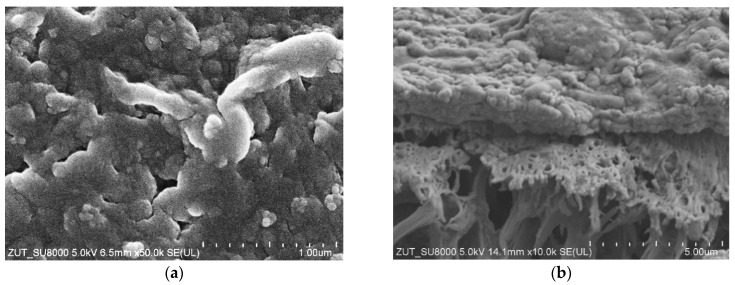
SEM image of FP100 membrane: (**a**) surface covered by deposit after 23 h of CWW1 separation (Figure 3b), and (**b**) membrane cross-section with deposit layer.

In UF plants operating at a constant capacity, the decrease in the permeate flux is prevented by raising the TMP value. In the case study, raising the TMP from 0.2 to 0.4 MPa increased the permeate flux to 113 LMH (Figure 3b). However, this value decreased quickly to 6 LMH within a few hours. After rinsing the membranes with water, a maximum permeate flux of 20 LMH was achieved. Moreover, the flux increased to only 47 LMH (W2) after washing the membranes with Euro Insect solution. Increasing the TMP to 0.4 MPa probably compressed the deposit layer on the membrane surface, making it difficult to remove [51]. SEM observations confirmed the formation of a compact fouling layer up to 2 µm thick on the surface of the membranes (Figure 4).

Washing efficiency is enhanced by raising the temperature of cleaning solutions to 323 K [12]. Indeed, washing the membranes with hot (323–328 K) Euro Insect solution improved cleaning efficiency, resulting in a maximum permeate flux of 120 LMH (Figure 3b, W3). However, the use of hot NaOH solutions should be limited, as they significantly accelerate membrane degradation [49]. Rapid PVDF degradation was observed when the temperature of the NaOH solution increased from 293 K to 343 K [3]. As demonstrated, the defluorination process increases rapidly with temperature [52]. For this reason, hot cleaning solutions were not used in subsequent UF studies. Furthermore, heating solutions complicate installation and generate additional costs, which is unacceptable for small car washes.

The obtained results suggest that using higher TMP values during the treatment of car wash wastewater is not favorable (Figure 3b). Similar conclusions were obtained in previous tests of FP100 membranes [50]. Therefore, the value of TMP was set to 0.1 MPa for further studies of the UF process. During overnight shutdowns, the wastewater remained in the installation, and the flux increased slightly when the plant was started up.

**Figure 5:**
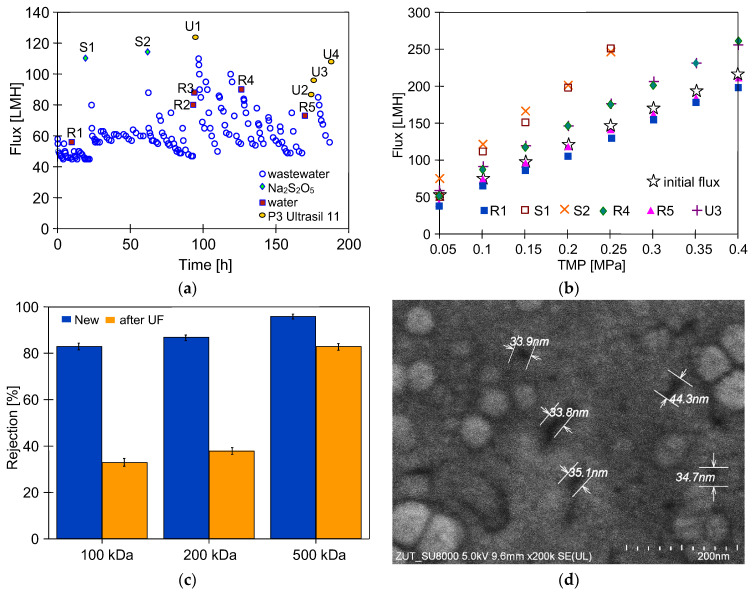
The results of membrane cleaning during CWW1 treatment: (**a**) changes in the permeate flux at TMP = 0.1 MPa, (**b**) changes in maximum permeate flux (initial flux—values determined for new FP100 membranes), (**c**) dextran rejection by new FP100 membranes and membranes after CWW1 filtration with cyclic chemical cleaning, (**d**) SEM images of FP100 surface after membrane chemical cleaning ((**a**), U4). R1–R5—membranes washed with water. S1, S2—module preserved with 0.25 wt% Na_2_S_2_O_5_ solution. U1–U4—membranes washed with 0.1 wt% P3 Ultrasil 11 solution (pH = 11.9).

The results obtained during the two-month operation of the UF system are shown in Figure 5a. Feeding the pilot plant with CWW1 decreased the permeate flux, which stabilized at 45 LMH. This value is similar to that obtained for TMP = 0.2 MPa (Figure 3b), which highlights the benefit of lowering the TMP value. Rinsing the system with water for one hour increased the permeate flux to 56 LMH (Figure 5a, R1). However, this value decreased quickly, as was also observed after subsequent water rinsing operations (R2–R5). This suggests that despite using a lower pressure (TMP = 0.1 MPa), a filtration cake forms on the membranes. Previous studies on CWW separation have shown that water rinsing does not completely remove this deposit [29,32]. A significant increase in the maximum permeate flux had been achieved after washing with the P3 Ultrasil 11 solution (Figure 5, U1–U4), which confirms that the alkali cleaner can effectively remove foulants and recover the permeate flux, as demonstrated in other studies [41].

Following the 22 h of CWW1 treatment (TMP = 0.1 MPa), the plant was preserved by rinsing with a 0.25% sodium metabisulfite solution for 30 min. The solution was then removed from the plant, and after a three-day break the UF testing was resumed. The installation was rinsed twice with water, and a maximum flux of 112 LMH was obtained (Figure 5a, S1). After a similar procedure following a five-day interruption, a maximum permeate flux of 115 LMH was achieved (Figure 5a, S2), indicating that the Na_2_S_2_O_5_ solution not only preserves the module but also possesses cleaning properties.

In aqueous solution, sodium metabisulfite hydrolyzes to form bisulfite ions (HSO_3_^−^), which act as strong reducing agents. This acidic environment facilitates the dissolution of mineral precipitates and promotes the partial hydrolysis of extracellular polymeric substances (EPS) within the fouling layer. These processes disrupt the structural integrity of the deposit matrix, enhancing its detachment from the membrane surface [53].

Effective membrane cleaning is indicated by the highest maximum permeate flux values after the S1 and S2 operations (Figure 5b). These values were significantly higher than the initial flux, indicating an increase in pore diameter due to repeated chemical cleaning (Figure 5a, points S and U). This conclusion is supported by the lower dextran rejection values obtained after studying the CWW1 treatment for two months (Figure 5c). A twofold better retention of 100 kDa dextran was obtained for the new membranes, which indicates that pores with sizes up to 10 nm predominated in these membranes [54,55,56]. Scanning electron microscopy (SEM) examination of the FP100 membranes performed after chemical cleaning revealed the formation of a small number of pores with diameters in the range of 30–45 nm (Figure 5d). These pores allowed feed to leak into the permeate, resulting in poorer dextran rejection.

### 3.3. Effects of Long-Term Alkaline Washing

**Figure 6:**
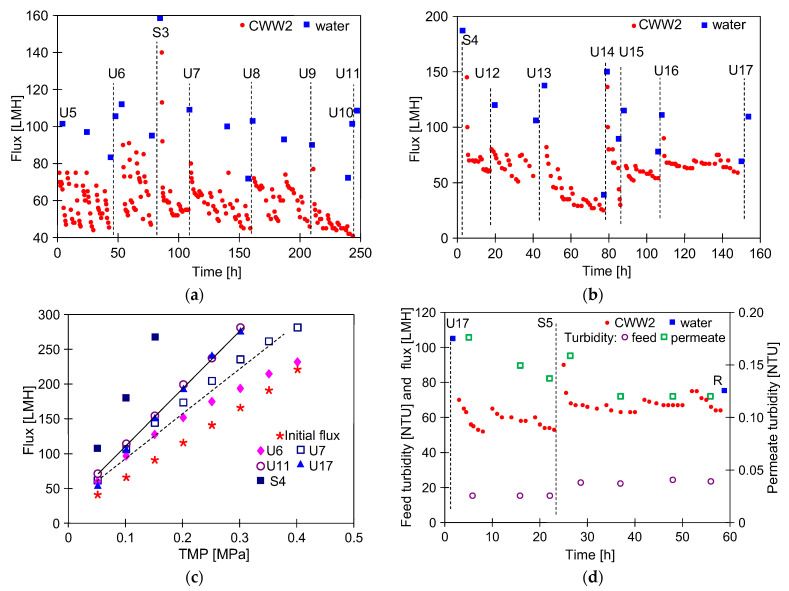
Effect of membrane washing with P3 Ultrasil 11 solution (U5–U17) during CWW2 treatment: (**a**,**b**) changes in permeate flux, (**c**) changes in maximum permeate flux, (**d**) changes in flux and turbidity. S3–S5—membranes preserved with 0.25 wt% Na_2_S_2_O_5_ solution. R—water rinsing.

The reduction in dextran rejection found after two months of CWW1 filtration (Figure 5c) raises questions about whether the FP100 membranes will deteriorate during prolonged UF operation with frequent alkaline cleaning. To better investigate washing effects, the membranes were chemically cleaned several times in the next stage of the study, using a 0.1 wt% solution of P3 Ultrasil 11 (pH = 11.9). For the UF tests, CWW2 was applied as the feed that filled the plant during the overnight break. The obtained values of the permeate flux are shown in Figure 6.

During the initial phase of CWW2 ultrafiltration (after U5), similar daily fluctuations in the permeate flux were observed. The next washing with a P3 Ultrasil 11 solution followed by a rinse with water increased the maximum permeate flux from 83 to 112 LMH (Figure 6a, U6). After preserving the membranes and taking a two-week break, the flux increased to 159 LMH (Figure 6a, S3). However, despite repeated alkaline cleaning, the permeate flux decreased to 40 LMH after 240 h of CWW2 filtration. Recovering the permeate flux to near its initial level (Figure 6a, U5) required twice-repeated chemical cleaning (U10 and U11). After these operations the UF installation was rinsed with the Na_2_S_2_O_5_ solution (S4), and was left unused for one month.

In the next period of CWW2 filtration the daily changes in the permeate flux (Figure 6b) were similar to those observed after maintenance S3 (Figure 6a). After chemical cleaning (U12), the maximum permeate flux was 110 LMH which was similar to the flux before the plant shutdown (Figure 6a, U11). This result suggests that maintenance and shutdowns of the UF plant do not significantly affect the performance of the tested PVDF membranes. However, the maximum permeate flux obtained after S4 preservation (Figure 6c, S4) was significantly higher than that measured after S1 and S2 preservation (Figure 5b). This indicates that larger pores were created in the membranes during the last few months of UF. Nevertheless, this process proceeded slowly, as indicated by the fact that a similar maximum permeate flux was obtained after U11 and U17 membrane washing (Figure 6c).

Washing water used at car wash stations with pressure guns must not contain suspended solids. UF tests have shown that, even with progressive membrane degradation, clean permeate with a turbidity of less than 0.2 NTU can still be obtained (Figure 6d). Chemical cleaning removes deposits from the membrane surface and unclogs a part of larger pores. This results in a slight increase in permeate turbidity (Figure 6d, 5 h). However, due to fouling, the larger pores quickly become blocked. This results in a decrease in the permeate flux from 72 to 55 LMH after washing U17 and a decrease in permeate turbidity from 0.18 to 0.13 NTU. As previously mentioned, maintaining the module with the Na_2_S_2_O_5_ solution promotes pore cleaning. Therefore, after S5 operation, permeate turbidity increased slightly before stabilizing at 0.12 NTU on subsequent UF testing days. These results suggest that the membrane-cleaning method used should not completely remove the fouling layer.

**Figure 7:**
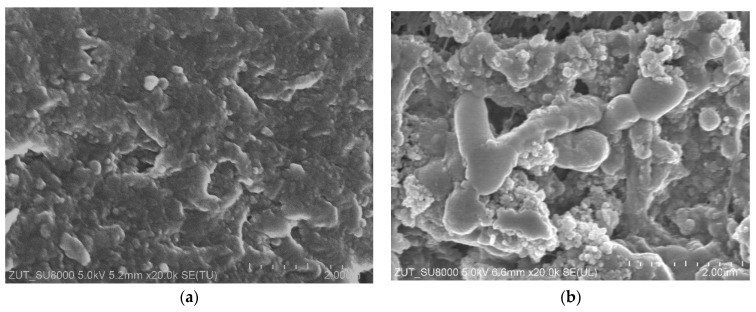
SEM images of FP100 membrane surface with deposit: (**a**) after U14 washing, (**b**) after U17 washing. Studies CWW2 filtration (Figure 6).

Although the UF process and the chemical cleaning of the membranes were repeated in a similar manner, the observed daily changes in the permeate flux during CWW separation often differed (e.g., Figure 6). SEM studies indicate that this may be due to differences in the degree of fouling layer removal. Figure 7 shows the FP100 membrane surface after U14 and U17 cleaning. Once the fouling layer had been mostly removed from the surface (Figure 7a), the CWW2 treatment led to deposit accumulation at the pore inlets, blocking the flow and resulting in a rapid decrease in the permeate flux (Figure 6b). After the U17 wash, some deposits remained on the membrane surface (Figure 7b), which resulted in a lower maximum permeate flux. However, new deposits were formed on the fouling layer, which protected the pores. As a result, the permeate flux was more stable (Figure 6d).

The UF pilot study conducted over several months showed that alkaline washing effectively reduced fouling caused by car wash wastewater. However, despite using the recommended chemical cleaning procedure for FP100 membranes, the repeated use of P3 Ultrasil 11 solution was found to increase the maximum permeate flux (Figure 6c). This indicates a significant increase in the number of large pores in the active layer of FP100 membranes. During CWW treatment, however, these pores quickly became blocked by the fouling layer (Figure 7), preventing a deterioration in the quality of the permeate obtained (Figure 6d, turbidity). However, in industrial applications, membranes should operate for at least two to three years. Therefore, over the following months, it was evaluated whether this performance could be maintained despite repeated alkaline washing.

**Figure 8:**
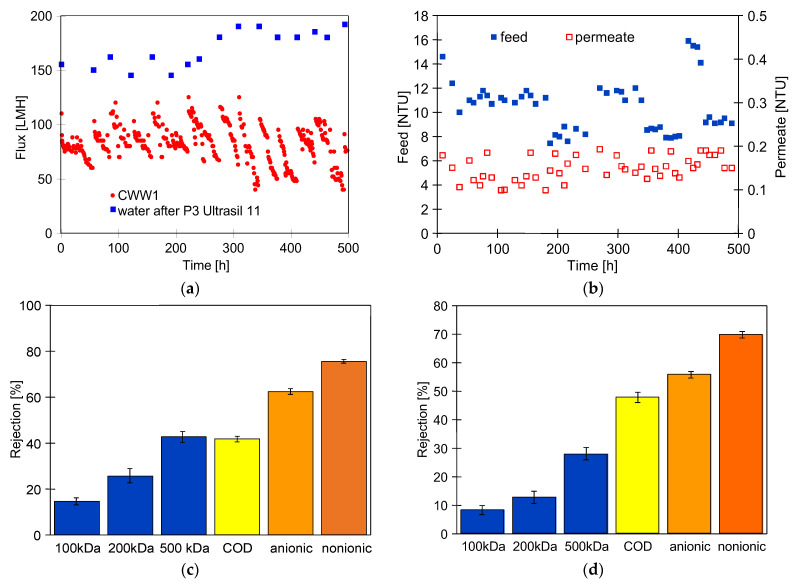
The influence of chemical cleaning (P3 Ultrasil 11 solution) on the UF process performance during CWW1 treatment: (**a**) changes in permeate flux, (**b**) changes in feed and permeate turbidity; and rejection of dextrans, anionic and nonionic surfactants, and COD determined after UF: (**c**) CWW2 (Figure 6), (**d**) CWW1 (**a**).

Figure 8 shows the results of over two months of UF plant operation, during which the membranes were chemically cleaned 16 times. As in previous UF tests, the module was filled with wastewater during overnight breaks. After the next one month of CWW1 treatment, the maximum permeate flux increased from 160 to 180 LMH (Figure 8a, 250 h). This indicates that the number of large pores in the membranes increased after washing with the P3 Ultrasil 11 solution. However, the turbidity tests showed that chemical cleaning had little effect on permeate quality. The measured turbidity values remained below 0.2 NTU throughout the study period (Figure 8b). Turbidity measurements were taken five hours after the membranes were chemically cleaned, allowing sufficient time for filtration cake formation. The deposition of feed components on the membranes reduces their permeability, but simultaneously repairs damage of the UF membrane separation layer [57].

The dextran separation studies confirmed that repeated chemical cleaning of the PVDF membranes increased pore size. As shown in Figure 8c, the dextran rejection values determined after the previous series of tests (Figure 6) were higher than those determined after the next 2 months of UF experiments (Figure 8d). Compared to the initial values shown in Figure 2, the rejection rate of 500 kDa dextran decreased from 95% to 30%. However, during CWW treatment, the retention of surfactants and COD did not deteriorate. This confirmed that the deposit formed on the membrane surface blocked the pores and improved the separation efficiency.

Various chemicals are used to wash cars, and they can affect the structure of the fouling layer during the separation of CWW [29,48]. Slight differences in the permeate flux decline were observed during the testing of CWW1 and CWW2 (Figure 6 and Figure 8). Cleaning agents may contain dyes (Table 1). It was found that the blue dye (5,5′-indigodisulfonic acid sodium salt) strongly adsorbed onto FP100 membranes, resulting in more severe fouling [32]. This may affect the effectiveness of membrane washing, as well as the membrane’s susceptibility to degradation by alkaline cleaning agents.

**Figure 9:**
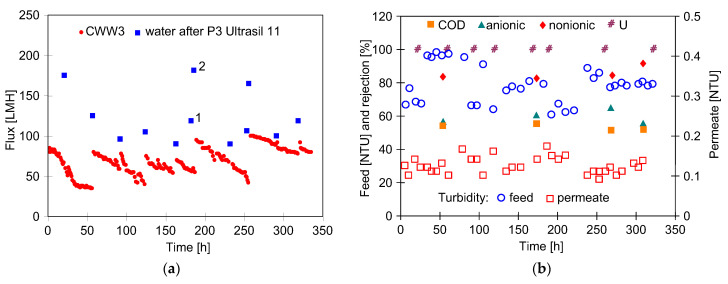
The influence of chemical cleaning (P3 Ultrasil 11 solution) on the UF process performance during treatment of CWW3 containing the blue dye: (**a**) changes in the permeate flux, (**b**) changes in turbidity of feed and permeate, and rejection of COD and surfactants (anionic and nonionic). U—membranes washed with P3 Ultrasil 11 solution.

To confirm the effectiveness of the alkaline membrane cleaning, a further experiment was conducted in which the UF plant was fed with CWW3 containing the blue dye. As expected, a greater decrease in the permeate flux was observed during CWW3 filtration (Figure 9a) compared to CWW1 and CWW2 (Figure 6a and Figure 8a). The efficiency of chemical cleaning also decreased, and after 90 h the maximum permeate flux stabilized at 100 LMH for CWW3 (with dye), while it was 180 LMH for CWW1 (without dye) (Figure 8a). This value was restored after the membranes were washed twice with P3 Ultrasil 11 solution (Figure 9a, points 1 and 2). This finding corroborates previous studies indicating that the adsorption of the blue dye onto the FP100 membranes impedes cleaning [32].

The more intense fouling may have resulted from the significantly higher turbidity of CWW3, reaching up to 100 NTU (Figure 9b). Nevertheless, the resulting permeate turbidity was below 0.2 NTU, similar to the other CWWs tested. The rejection of COD was approximately 52%, while that of anionic surfactants was about 60%. These values are similar to those determined previously (Figure 8d), indicating that the presence of the blue dye did not affect the rejection of CWW3 components.

### 3.4. Effects of Membrane Cleaning with 0.5 Vol.% Euro Wheel Cleaner

The high efficiency of chemical cleaning during the CWW1-CWW3 separation process confirmed the effectiveness of P3 Ultrasil 11, a cleaning agent recommended by the FP100 membrane manufacturer. Previous studies have demonstrated that fouling during CWW separation can be mitigated by employing wheel cleaners (Euro Wheel Cleaner) and insect removers (Euro Insect), which are commonly used in car washes [29,48]. These cleaning solutions have a similar composition to the P3 Ultrasil 11 solution (Table 1). Therefore, in the next stage of the pilot study the suitability of the Euro Wheel Cleaner solution for membrane cleaning during the separation of CWW4, which contains blue dye, was assessed.

**Figure 10:**
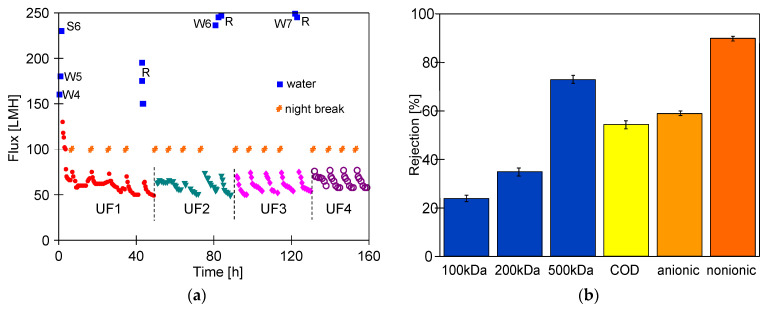
Effect of membrane cleaning with 0.5 vol.% Euro Wheel Cleaner (EWC) on: (**a**) permeate flux during CWW4 separation, (**b**) rejection after UF4 series. S6—membrane preservation with Na_2_S_2_O_5_ solution, W4–W7—EWC washing (30 min). R—water rinsing. UF1 series—module filled with CWW4 during overnight break; membranes subsequently washed with EWC (30 min). UF2 series—membranes washed with EWC (30 min) after CWW4 filtration, module filled with EWC during overnight and weekend break; UF3 series—similar to UF2, but installation filled with water over the weekend; UF4 series—similar to UF1, but washing extended from 30 to 60 min.

The study began with double membrane washing, which increased the maximum permeate flux from 120 to 180 LMH. After preserving the plant for one week, the flux increased to 230 LMH (Figure 10, S6). This result suggests that the majority of the deposit formed during CWW3 filtration (Figure 9) was removed from the membranes. After feeding the pilot plant with a 0.5% Euro Turbo Foam Color Blue solution, the permeate flux decreased to 100 LMH. Then, 0.2 vol.% Euro Blue Wax was added to the feed, which creates CWW4. After adding the wax, the permeate flux decreased to 68 LMH within a few minutes and then stabilized at 57 LMH. These results confirm the previous reports indicating that hydrowaxes used for car washes significantly increase fouling during CWW separation [58].

The efficiency of the removal of the fouling layer from membranes was evaluated using four cleaning methods (Figure 10a). First, after the UF process, CWW4 was left in the plant overnight. The module was then flushed with water (100 L) and washed with Euro Wheel Cleaner (EWC) solution for 30 min (UF1 series). Subsequently, CWW4 filtration was resumed. Consequently, after four days, the UF permeate flux decreased to 50 LMH, and the following day it dropped further to 46 LMH. This indicated the build-up of irreversible fouling.

In the second method, after 6–7 h of CWW4 filtration, the UF plant was rinsed with Euro Wheel Cleaner solution for 30 min, and the cleaner was left in the plant overnight (UF2 series). After the overnight break, the module was flushed with 100 L of water, and CWW4 separation was resumed. This method kept the permeate flux stable for five consecutive days. However, the maximum permeate flux increased to 250 LMH (Figure 10a, 80 h), which indicates that this solution has one disadvantage: the long contact time (weekend) between the membranes and the alkaline solution can accelerate PVDF degradation. Therefore, in the third method, the module was filled with water during the weekend break (UF3 series). Consequently, the maximum permeate flux remained at 250 LMH.

In the fourth method, the CWW4 filled the UF system overnight. The membrane was subsequently washed with EWC for 60 min. Extending the washing period resulted in a permeate flux of 55 LMH for the subsequent week of testing (Figure 10a, UF4 series).

The UF experiments showed that the daily use of the alkaline agent typically employed for car washing to clean the membranes did not cause a significant increase in the degradation of the tested PVDF membranes. In each case studied, permeate with a turbidity of less than 0.2 NTU was obtained. Tests performed after completing the UF4 series for separating CWW components (Figure 10b) revealed comparable COD and surfactant rejection to that observed in previous studies (Figure 8d). Additionally, significantly higher dextran retention was achieved due to the membranes being covered with a fouling layer, unlike in previous tests (Figure 8). The results indicate that 27 months of PVDF membrane operation did not affect the quality of the permeate obtained from the tested CWW.

### 3.5. Membrane Degradation

Periodic analyses of dextran separation conducted during the study revealed a gradual decline in dextran rejection (Figure 2, Figure 5 and Figure 8). These studies were conducted using thoroughly washed membranes and demonstrated that alkaline treatment of PVDF membranes alters the pore structure and enlarges the pores, as indicated by the results of other studies [41,49]. Changes in surface structure were observed each day during the soaking of PVDF in NaOH solutions [40]. However, the results of ongoing CWW separation studies also indicate stabilization periods relating to changes in FP100 membrane properties (e.g., Figure 8a).

**Figure A2:**
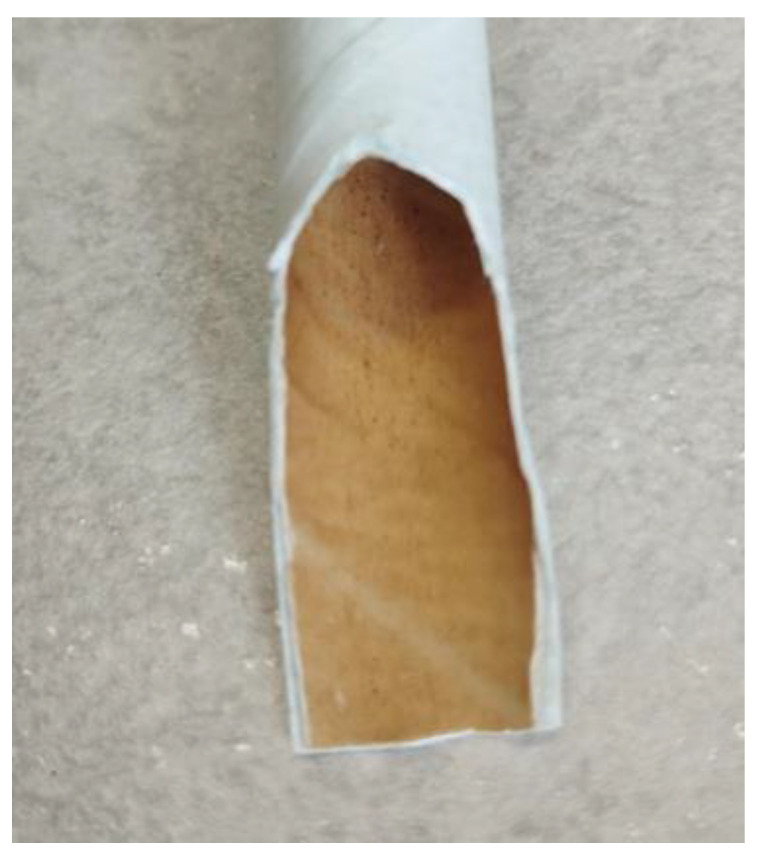
Internal view of the FP100 membrane after completion of 27 months of UF operation.

**Figure A3:**
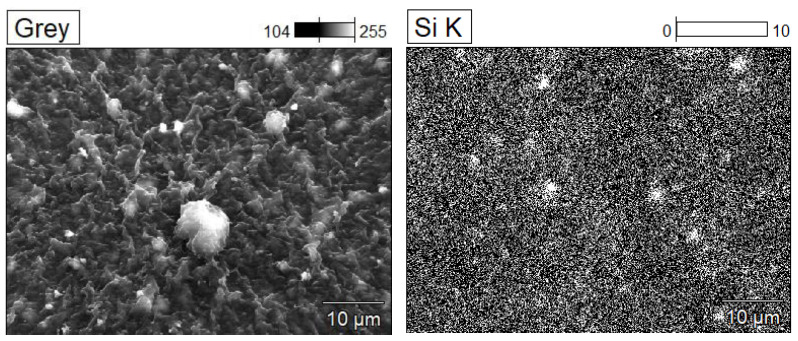

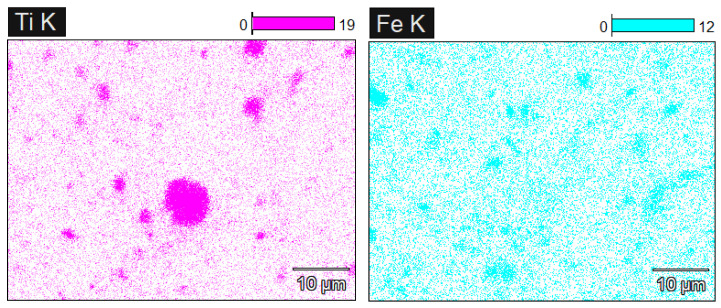
SEM image of deposits formed on the membrane surface after 27 m UF process studies and results of SEM-EDS analysis.

After completing the UF experiments, it was found that the membrane surfaces were covered with a brown-colored deposit (Figure A2). The intensity of this coloration, regardless of the position of the tubes in the housing of module B1, was similar for all membranes. SEM-EDS analysis showed that, in addition to C, the deposit also contained significant amounts of Si, Ti, and Fe (Figure A3).

**Figure 11:**
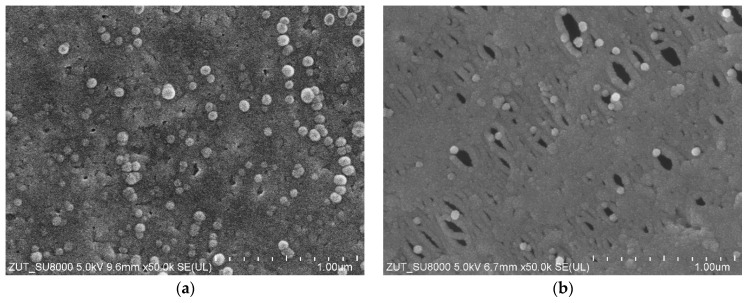
SEM images of FP100 membrane surface: (**a**) new membrane, (**b**) membrane after long-term (27 months) filtration of CWW with periodic alkaline cleaning.

The deposit was removed from the membranes using an HCl solution, and SEM analysis performed on the cleaned membrane confirmed that significantly larger pores had developed in the membranes during pilot-scale operation (Figure 11). The appearance of pores in the range of 100–300 nm accounted for the deterioration observed in dextran separation. Similar effects were reported in our previous study, in which pores with sizes of 100–200 nm were observed after an 18-month UF operating period [39]. However, the present SEM analyses demonstrated that membrane washing with the alkaline agents used for car washing caused further enlargement of the largest pores, increasing their size from approximately 200 nm to about 300 nm during the additional 9 months of operation (Figure 11b).

As shown in Figure 7 and Figure A2, the applied membrane-cleaning methods did not completely remove the deposit from the membrane surfaces. As a result, during CWW treatment, the fouling layer formed on the membrane surface effectively blocked these enlarged pores. As a result, despite more than 27 months of continuous module operation, the membranes showed no significant decline in the removal of turbidity, COD, or surfactants (Figure 9b and Figure 10b).

Numerous spherical agglomerates were observed on the surfaces of the new membranes, whereas their quantity was significantly reduced after 27 months of operation (Figure 11). Similar spherical agglomerates on the surfaces of polyethersulfone membranes have been reported to originate from polyvinylpyrrolidone (PVP), which is added to enhance the membrane’s hydrophilicity [59]. It is therefore likely that PVP was also incorporated into the FP100 membranes. FTIR analyses of the membrane samples shown in Figure 11 revealed characteristic PVP peaks (Figure A3) at 1386 cm^−1^ (C–N) and 1635–1695 cm^−1^ (C=O amide bond) [57,60,61]. The intensity of these peaks decreased markedly in the samples collected after 27 months of UF operation, confirming previous findings indicating a rapid leaching of PVP from membranes when exposed to alkaline cleaning agents [59].

**Figure 12:**
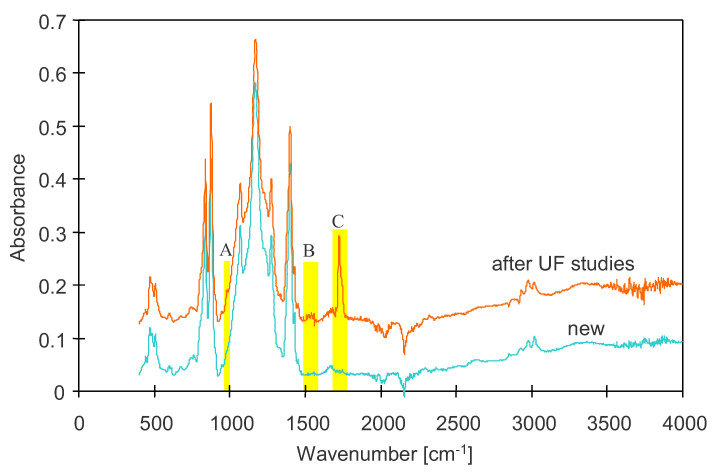
ATR-FTIR spectra of new PVDF membrane (Figure 11a) and sample collected from UF pilot plant after completion of long-term (27-month) CWW filtration with cyclic alkaline cleaning. Prior to FTIR analysis, the residual deposit was removed from the membrane surfaces (Figure 11b). A, B, C—new peaks indicating degradation.

In addition to PVP leaching, the formation of large pores resulted from a degradation of the membrane matrix, which was also confirmed by the FTIR analyses. The action of NaOH solutions can alter the structure of PVDF membranes and reduce polymer crystallinity, which can significantly impact their performance [3,42]. The FTIR spectrum of the studied membranes (Figure 12) shows the appearance of bands attributed to the α and β phases of PVDF. Absorption bands at 762, 796, 841, 873, 974, 1066, 1178, and 1423 cm^−1^ are characteristic of the α phase [41,49]. The remaining bands at 841, 1182, 1274, and 1403 cm^−1^ correspond to the β phase of PVDF [5,49]. The intensity of these peaks decreased after the alkaline cleaning, as was also observed in other studies [4,44]. In contrast, the positions of these peaks remained unchanged, and only one additional small peak indicating an α phase was detected in the washed FP100 membrane sample (Figure 12, A). This suggests that there were no significant changes in the crystalline structure of the PVDF membrane matrix. In another paper, the main peaks of PVDF also did not change significantly after long-term chemical cleaning [40]. In contrast, noticeable alterations were observed in another investigation following a 5-day immersion of the PVDF membrane in a 2.5% NaOH solution [4].

The FTIR spectra of the membrane samples after operation in a UF pilot plant showed two new peaks at 1540–1590 cm^−1^ and 1750 cm^−1^ (Figure 12, B and C), which are indicative of the dehydrofluorination process [3,30,49]. The dehydrofluorination effect was caused by NaOH treatment, which led to the formation of C=C bonds. In another study [62], the dehydrofluorination effect induced by NaOH was attributed to the peak appearing at 1729 cm^−1^. The presence of peaks in the 1590–1650 cm^−1^ range, attributed to a carbon–carbon double bond, and in the 1700–1800 cm^−1^ range, associated with carbonyl groups, was also reported as a result of alkaline washing [30,44,49].

## 4. Conclusions

Alkaline cleaning agents effectively restored the performance of tubular PVDF membranes fouled during the ultrafiltration of treated car wash wastewater (CWW). Throughout several months of pilot-scale operation and despite considerable variability in CWW composition, the tested UF module consistently produced permeate with turbidity below 0.2 NTU and achieved COD and surfactant rejection exceeding 50%.

In addition to professional membrane-cleaning agents such as P3 Ultrasil 11, the alkaline detergents commonly used in car wash facilities were also shown to be suitable for maintaining PVDF membrane performance. Stable operation was achieved when cleaning was performed every 7–10 h, with an alkaline washing step lasting at least 30 min and preferably 60 min.

The long-term study confirmed that PVDF membranes are not fully resistant to alkaline solutions. After 27 months of pilot-scale operation, extensive structural changes were observed, including the formation of numerous enlarged pores (100–300 nm) and FTIR analysis confirmed the dehydrofluorination of the polymer matrix. Nevertheless, these structural changes did not compromise the permeate quality. The enlarged pores became rapidly occluded during filtration, and the resulting fouling layer—although reducing the permeate flux—provided effective secondary separation, compensating for the loss of intrinsic membrane selectivity.

Overall, the findings demonstrate that economically accessible cleaning agents used in car wash facilities can sustain membrane functionality over extended periods, even under conditions that promote PVDF aging. The results highlight both the operational feasibility of low-cost cleaning strategies and the long-term structural limitations of PVDF membranes in car wash water reuse systems.

During short installation shutdowns, treated CWW can safely remain inside the system without causing additional fouling or membrane degradation. For shutdowns of more than two days, however, the system should be flushed with water and the membranes preserved with a 0.25% Na_2_S_2_O_5_ solution.

## Figures and Tables

**Table 1:** Composition of cleaning agent concentrates.

Agent	Component	[wt%]
Euro Turbo	Diethylene glycol butyl ether	1–5
Foam	Benzenesulfonic acid, 4-C10-13-sec-alkane, sodium salts	1–5
	polymers	1–3
		
Euro Turbo	Benzenesulfonic acid, 4-C10-13-sec-alkane, sodium salts	1–5
Foam	Green dye	-
Color Green	Diethylene glycol butyl ether	1–5
	polymers	1–3
		
Euro Turbo	5,5′-indigodisulfonic acid sodium salt	-
Foam	Diethylene glycol butyl ether	1–5
Color Blue	Benzenesulfonic acid, 4-C10-13-sec-alkane, sodium salts	1–5
	polymers	1–3
		
Hydrowax	Diethylene glycol monobutyl ether	10–20
	polymeric waxes	1–2
		
Euro Blue	5,5′-indigodisulfonic acid sodium salt	-
Wax	Diethylene glycol monobutyl ether	10–20
	polymeric waxes	1–2
		
Euro Wheel	NaOH	3–5
Cleaner	Ethylenediaminetetraacetic acid tetrasodium salt	3–5
	Sulfonic acids, C14-16-alkane hydroxy and C14-16-alkene, sodium salts	5–7
	Diethylene glycol butyl ether	3–5
	1-Propanaminium, 3-amino-N-(carboxymethyl)-N,N-dimethyl-, N-(C12-18(even numbered) acyl) derivs	0.5–1.5
Euro Insect	NaOH	3–5
	Ethylenediaminetetraacetic acid tetrasodium salt	3–5
	Sulfonic acids, C14-16-alkane hydroxy and C14-16-alkene, sodium salts	5–7
	Diethylene glycol butyl ether	3–5
P3 Ultrasil 11	NaOH	>40
(powder)	Ethylenediaminetetraacetic acid tetrasodium salt	>30
	anionic surfactants	<5
	non-ionic surfactants	<5

**Table 2:** Parameters of prepared synthetic CWW. Foam solution: A—Euro Turbo Foam, B—Euro Turbo Foam Color Green, C—Euro Turbo Foam Color Blue. Waxes: H—Hydrowax, EB—Euro Blue Wax. COD—chemical oxygen demand. Anionic, nonionic—surfactants.

CWW	Agents	Turbidity [NTU]	COD [mg/L]	Anionic [mg/L]	Nonionic [mg/L]
CWW1	A-H	15.7 ± 3.8	2249 ± 105	432 ± 20	15.9 ± 3
CWW2	B-H	17.6 ± 5.1	2332 ± 129	440 ± 18	16.2 ± 3
CWW3	C-EB	121 ± 31.4	2936 ± 265	414 ± 32	34.2 ± 9
CWW4	C-EB	232 ± 48.2	3296 ± 165	404 ± 23	25.3 ± 8

**Figure A4:**
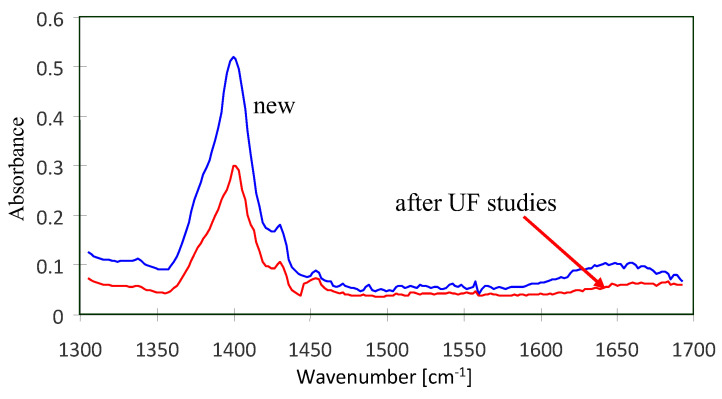
ATR-FTIR spectra of the new FP100 membrane and the membrane sample collected from the UF pilot plant after completion of long-term (27-month) CWW filtration with cyclic alkaline cleaning.

## Data Availability

The original contributions presented in the study are included in the article; further inquiries can be directed to the corresponding author.

## Appendix A

Module B1 contains 18 tubular membranes arranged around a central pipe on the sides of hexagonal structures (Figure A1). For SEM analyses, one tube from the inner hexagon (starting from the top) was removed and replaced with a new membrane. Membrane samples for examination were collected 50–60 cm from the tube inlet. To remove any remaining feed solution, the samples were rinsed with distilled water. To visualize changes in the structure of the skin layer (Figure 11b), the deposit on the membrane surfaces was removed using a HCl solution.

After completion of the UF experiments, all tubes installed in module B1 exhibited a brown coloration on their membrane surfaces (Figure A2).

Figure A3 presents the SEM image of the membrane surface covered with the deposit (grey) along with the SEM-EDS analysis of the deposited layer.

The white color of the membranes was restored by removing the brown deposit from their surfaces using a HCl solution. The ATR-FTIR spectra of the cleaned and the new membrane are presented in Figure A4.
